# Diabetic Kidney Disease Update

**DOI:** 10.1111/1753-0407.70150

**Published:** 2025-09-05

**Authors:** Christian Mende, Zachary Bloomgarden

**Affiliations:** ^1^ Department of Medicine University of California San Diego La Jolla California USA; ^2^ Department of Medicine, Division of Endocrinology, Diabetes and Bone Disease Icahn School of Medicine at Mount Sinai New York New York USA

The major determinants of the development of chronic kidney disease (CKD) in people with diabetes are hyperglycemia, hypertension, genetic susceptibility, dyslipidemia, and inflammation. By better understanding these factors, we can modify the risk of kidney damage and subsequent complications among people with diabetes. Elevation in glucose levels leads to both metabolic and hemodynamic changes, including glomerular hyperfiltration, podocyte injury, and progressive albuminuria, while hypertension accelerates glomerular damage [1]. Genetic predisposition, along with lifestyle factors such as obesity and smoking, further increases the risk. Dyslipidemia and oxidative stress contribute to endothelial dysfunction and tubulointerstitial injury [2], and inflammation [3] and activation of fibrotic pathways play important roles in disease progression [4, 5].

CKD is defined operationally by estimated glomerular filtrate rate (eGFR) < 60 mL/min and urine albumin/creatinine ratio (UACR) ≥ 30 mg/g present for 90 days or longer. Diabetes is responsible for roughly one‐quarter to one‐half of all CKD cases, with the proportion varying by region, population demographics, and stage of kidney disease [6, 7, 8]. This underscores the critical importance of diabetes prevention and optimal management to reduce the global burden of CKD. Albuminuria with normal renal function and/or an eGFR < 60 mL/min is associated with considerable cardiovascular mortality and heart failure risk. This has been underappreciated compared to the risk of progression of CKD and ESKD. In diabetic CKD, the risk of cardiovascular death is twice as great with eGFR < 60 mL/min and four times as great with eGFR < 45 mL/min, compared to normal renal function [9]. Compared to no albuminuria, mortality and heart failure admissions are four‐ and five‐fold more likely in the presence of albuminuria, even when the eGFR is normal [10, 11].

Pharmacologic therapy is the cornerstone of treatment of diabetic CKD, with improvement in outcome seen with Renin‐Angiotensin‐Aldosterone system (RAAS) blockade, including mineralocorticoid inhibitors (MRA), sodium glucose transporter (SGLT) 2 inhibitors, and glucagon‐like peptide (GLP)‐1 receptor agonists (RA); significant therapeutic benefits also have been shown with aggressive therapy of comorbidities (hypertension, obesity and dyslipidemia) [12]. However, lifestyle modifications (diet/weight management, physical activity, smoking) and genetic risk have not received as much attention in clinical practice [13]. In a Dutch observational study, only 2% of patients adhered to all recommended lifestyle recommendations [14].

Three publications in the current Journal of Diabetes evaluate the aspects of the effects of lifestyle, social factors, genetic risks, and comorbidities as risk factors for the progression of CKD and the development of end‐stage kidney disease (ESKD). Cui and coworkers use longitudinal data from the China Health and Retirement Longitudinal Study (CHARLS) of 93,226 participants followed from 2011 to 2020 to examine social determinants and lifestyle factors associated with self‐reported diabetes and kidney disease among Chinese aged 45 and older. The authors found a 10‐fold increase from 2011 to 2020, particularly in males, with the particularly great increase in 2020 potentially related to the additional impact of the COVID pandemic [15]. They cite factors including greater age and reduced access to healthcare in urban areas, with the highest CKD incidences in the northern regions of the country. Of note, lifestyle choices such as smoking, physical inactivity, and poor diet appear from their analysis to be mostly responsible. Recommendations include urgently addressing positive lifestyle factors and social determinants as critical needs to reduce the risk of diabetic CKD.

In a second study, Wang and coworkers study UK Biobank participants to assess the joint contributions of genetic risk and lifestyle factors in the progression of diabetes to diabetic nephropathy using prospective data comparing 1335 persons with diabetes who progressed to diabetic nephropathy with 10,646 persons with diabetes not showing such progression. Lifestyle factors analyzed included BMI, smoking status, alcohol consumption, and self‐reported diet and physical exercise, while genetic susceptibility to CKD was based on a previously validated polygenic risk score. Age, longer diabetes duration, higher HbA1c, and hypertension were associated with risk, as were lower educational and income status; the benefit of more favorable lifestyle factors was particularly seen in participants with high genetic risk, suggesting an approach that might allow stratification of individuals with the greatest likelihood of benefit from lifestyle interventions [16].

Liu and coworkers analyzed three cohorts of persons with diabetes to assess the question of whether higher circulating ketone levels might be associated with better renal outcome [17]. In the National Health and Nutrition Examination Survey (NHANES) database, 1257 people with diabetes had CKD, with a dietary ketogenic ratio calculated from two 24‐h diet recalls based on the ratio of fat plus protein intake to total nutrient intake showing a trend to greater likelihood of ESKD with lower dietary ketogenic index. In a longitudinal study of 346 patients with diabetes and biopsy‐documented diabetic kidney disease, with median urinary protein output 5.0 g/day, there was progressively lower likelihood of adverse renal outcome with increasing β‐hydroxy butyrate (B‐OHB), the highest quartile of B‐OHB, 0.28–0.99 mM/L, having just over half the rate of renal disease progression seen in those with the lowest quartile (> 0.08 mM/L) over a 27‐month period of observation. Finally, genetic variants associated with higher 3‐hydroxybutyrate used in a Mendelian Randomization study suggested a significant inverse association of B‐OHB with serum cystatin C and creatinine levels. It is noteworthy that a consistent action of SGLT2 inhibitors is to increase B‐OHB, an effect which has been hypothesized to contribute to their protective benefit [18].

What clinical message can one derive from the three discussed publications? The epidemiologic evidence of benefit of lifestyle intervention is in accord with the 2024 KDIGO CKD guidelines for slowing diabetic CKD in control of hypertension (BP < 130/80 mmHg with systolic > 120 if tolerated), dyslipidemia (LDL < 70 mg/L), obesity (BMI ≤ 27) and hyperglycemia (HbAIC < 7%) [12].

More attention is needed to dietary modification with protein restriction favoring plant proteins and lower salt intake, to encouraging regular exercise, to not smoking, and to increasing physical activity to at least 150 min/week. The effect of SGLT2 inhibitors in increasing ketone levels may be of greater importance than generally recognized, and appropriate dietary modification to safely accomplish this without causing ketoacidosis may be a goal of future studies. The findings of incremental improvement in favorable lifestyle being associated with reduction in diabetic CKD are impressive and enforce its importance. Finally, the role of genetic factors in diabetic CKD is beginning to be better understood, and studies of this aspect of CKD prevention should be encouraged.

## Conflicts of Interest

The authors declare no conflicts of interest.
